# Multi-platform evidence of GSDMD-dependent pyroptosis in malaria with a potential role for TLR7

**DOI:** 10.3389/fimmu.2026.1862560

**Published:** 2026-08-26

**Authors:** Yumei Gong, Yiwei Liu, Longkun Li, Jialin Geng, Ying Huang, Lixin Luo, Xinyue Wang, Long Xu, Yongtao Zhang, Xingfei Pan, Lu Li, Shan Zhao, Jun Huang

**Affiliations:** 1 Department of Infectious Diseases, Guangdong Provincial Key Laboratory of Major Obstetric Diseases, Guangdong Provincial Clinical Research Center for Obstetrics and Gynecology, The Third Affiliated Hospital, Guangzhou Medical University, Guangzhou, China; 2 Key Laboratory of Immunology, Sino-French Hoffmann Institute, School of Basic Medical Sciences, Guangzhou Medical University, Guangzhou, China; 3 Department of Laboratory Medicine, The Sixth Affiliated Hospital of Guangzhou Medical University, Qingyuan People’s Hospital, Qingyuan, China; 4 Guangdong Provincial Key Laboratory of Allergy and Clinical Immunology, the Second Affiliated Hospital, Guangzhou Medical University, Guangzhou, China

**Keywords:** GSDMD, immunomodulation, macrophage, malaria, pyroptosis, TLR7

## Abstract

**Background:**

Macrophage-mediated inflammation critically determines malaria pathology. Although pyroptosis contributes to host defense, excessive activation induces severe immunopathology. The precise pyroptotic pathways in splenic macrophages during Plasmodium infection, and the upstream regulatory mechanisms that govern them, remain unclear.

**Methods:**

We integrated clinical transcriptomic analysis (GSE132050), *Plasmodium yoelii* infected mouse models, and *in vitro* macrophage assays to systematically investigate pyroptosis mechanisms and to evaluate the regulatory role of the Toll-like receptor 7 (TLR7) agonist R848.

**Results:**

Clinical data revealed significant up-regulation of core pyroptosis genes (GSDMD, CASP1/4/5) in malaria patients, correlated with myeloid expansion. *In vivo*, *P. Yoelii infection* was associated with activation of both canonical and non-canonical gasdermin D (GSDMD) related pyroptotic signaling in CD11b^+^F4/80^+^ splenic macrophages. *In vitro*, infected red blood cell (iRBC) lysates directly triggered post-translational cleavage of Caspase-1/11 and GSDMD, causing rapid membrane perforation and inflammatory efflux. Notably, *in vivo administration* of R848 significantly suppressed this pyroptotic activation, exerting a particularly strong inhibitory effect on the Caspase-11/GSDMD non-canonical pathway.

**Conclusions:**

Plasmodium infection is associated with canonical and non-canonical pyroptosis in splenic macrophages. R848 treatment was associated with reduced pyroptotic signaling in splenic macrophages, suggesting a regulatory role for TLR7.

## Introduction

Malaria is a severe mosquito-borne infectious disease caused by Plasmodium infection, with severe malaria caused by Plasmodium falciparum accounting for more than 99% of global malaria-related deaths. According to the World Health Organization (WHO) World Malaria Report 2023, there were approximately 263 million malaria cases globally in 2023, resulting in about 597,000 deaths, with an overall upward trend over the past decade (1, 2). As Plasmodium parasites develop increasing resistance to traditional first-line antimalarial drugs, such as chloroquine and artemisinin, the urgent need for new intervention targets from the perspective of host immune regulation, as well as alternative solutions beyond direct insecticides, has become a major focus in current malaria prevention and treatment research (3–5). In this process, the spleen, a core immune organ in the body’s defense against malaria, plays a critical role through macrophages, which phagocytose and eliminate infected red blood cells to regulate parasite burden. The inflammation mediated by these macrophages directly determines the pathological outcome of malaria.

In the host defense against pathogens, pyroptosis, a key form of programmed cell death, plays a crucial role (6). Pyroptosis is executed by the GSDMD protein and includes two major pathways: the canonical pathway, which relies on inflammasome sensors, such as absent in melanoma 2 (AIM2) and interferon gamma-inducible protein 16 (IFI16) to recognize pathogen-associated molecular patterns (PAMPs) and activate Caspase-1, and the non-canonical pathway, in which cytosolic pathogen components directly activate human Caspase-4/5 or mouse Caspase-11. Both pathways ultimately lead to GSDMD cleavage, forming membrane pores that induce pyroptosis and the massive release of inflammatory factors. Moderate pyroptosis can eliminate intracellular niches for pathogen replication, whereas excessive pyroptosis can lead to uncontrolled inflammation and tissue damage. Previous studies have shown that parasite infections, such as Leishmania and Giardia can activate the pyroptosis pathway (7, 8). In malaria, *Plasmodium falciparum* infection can induce GSDMD-dependent pyroptosis in hepatocytes (9). However, current research on pyroptosis in malaria has largely failed to differentiate its subtypes (10), and the specific molecular mechanisms by which Plasmodium infection induces pyroptosis in splenic macrophages remain unresolved. Without a systematic understanding of the specific contributions of canonical and non-canonical pyroptotic pathways in splenic macrophages, potential therapeutic targets for intervening in severe malaria-induced pathological damage may be missed.

Toll-like receptors (TLRs) are key regulators of innate immune recognition, and their signaling is often closely coupled with inflammasome activation and pyroptosis (11, 12). Existing studies have shown that TLR2, TLR4, and TLR9 play important roles in the early recognition of Plasmodium components (13). Particularly, TLR9, which can be activated by histidine-rich glycoproteins, initiates the myeloid differentiation primary response 88 (MyD88)-dependent inflammatory pathway and directly mediates immune defense during the hepatic stage of Plasmodium infection (14–16). Given that TLR7 and TLR9 are both endosomal pattern recognition receptors for nucleic acids and share highly similar functional characteristics, we hypothesize that TLR7 may also be involved in a similar immune regulatory network. However, there have been no systematic reports on how TLR7 regulates macrophage pyroptosis during *Plasmodium* infection, especially its effects on GSDMD activation and downstream inflammatory cascades.

This study integrates clinical whole-blood transcriptomic bioinformatics analysis, *Plasmodium yoelii (P. yoelii*, non-severe malaria)- infected mouse models, and *in vitro* macrophage stimulation experiments, aiming to elucidate the intrinsic connection between Plasmodium infection and pyroptosis in splenic macrophages and to evaluate the regulatory potential of TLR7. The specific research is conducted at three levels.

## Methods

### Experimental animals and ethics

Specific-pathogen-free (SPF) female C57BL/6 wild-type mice (6–8 weeks old) were purchased from Guangdong Zhiyuan Biological Technology Co., Ltd. TLR7 whole-body knockout (TLR7^-^/^-^) mice were obtained from The Jackson Laboratory. Plasmodium *yoelii*(NSM strain) was supplied by the Malaria Research and Reference Reagent Resource Center. All animal procedures were approved by the Institutional Animal Care and Use Committee of Guangzhou Medical University (approval No. S2024-282) and conducted in accordance with the guidelines for the care and use of laboratory animals. Human data ethics statement. The clinical transcriptomic dataset GSE132050 was obtained from the publicly available Gene Expression Omnibus (GEO) database (https://www.ncbi.nlm.nih.gov/geo/query/acc.cgi?acc=GSE132050). As this study represents a secondary bioinformatics analysis of publicly available, de-identified data, no additional ethical approval or informed consent is required under the guidelines of our institution and the Declaration of Helsinki.

### Establishment of *Plasmodium* infection and drug intervention

Frozen Plasmodium-infected red blood cells (iRBCs) stored at –80 °C were thawed at 37 °C and inoculated into C57BL/6 mice for parasite revival and expansion. When parasitemia reached 10%–20%, the iRBCs were diluted in sterile phosphate-buffered saline (PBS) to 1×10^7^ cells/mL, and each mouse was injected intraperitoneally with 100 µL of the suspension to establish infection. For the R848 intervention, treatment was initiated on day 6 post-infection (when parasitemia reached approximately 5%). Mice in the intervention group received intraperitoneal injections of R848 (100 µg per mouse) every other day for a total of three injections (cumulative dose of 300 µg per mouse), while control mice received an equal volume of sterile PBS. On day 12 post-infection, mice were sacrificed by cervical dislocation, and spleens were aseptically harvested for subsequent experiments.

### Bioinformatics analysis of clinical transcriptomic data

The whole-blood transcriptomic dataset GSE132050 was downloaded from the Gene Expression Omnibus (GEO) database, which includes time-series samples from 15 patients with Plasmodium falciparum and 4 healthy controls (17). Data processing, differential expression analysis, Kyoto Encyclopedia of Genes and Genomes (KEGG) pathway enrichment, and gene set enrichment analysis (GSEA) were performed using R software (version 4.2.0). The limma package was used for background correction and normalization; genes with |log_2_(fold change)| > 0.5 and *P* < 0.05 were defined as differentially expressed. KEGG and GSEA were performed using the clusterProfiler and GseaVis packages. Immune infiltration was computationally inferred using the CIBERSORT deconvolution algorithm (18). Correlations were assessed using Spearman (immune cell proportions) or Pearson (TLR7 vs pyroptosis genes) coefficients; the pyroptosis gene set was obtained from MSigDB (REACTOME_PYROPTOSIS).

### Cell and molecular biology experiment preparation of splenic single-cell suspensions

Mouse spleen tissues were gently ground on a 200-mesh sterile nylon filter in D-Hank’s solution. After red blood cell lysis, the cells were centrifuged and re-suspended to generate splenic single-cell suspensions. The cell content was counted under a microscope, and the cell concentration was adjusted to 2 ×10^6^/mL.

### Flow cytometry analysis

Single lymphocytes isolated from spleen were stained with different fluorescence labeled antibodies against mouse surface markers (CD11b, F4/80), at 4 °C for 30 minutes, in dark. Then cells were treated with a transcription factor staining kit (00-5523-00, Invitrogen, USA) and stained with fluorescent antibodies for intracellular proteins (TLR7, Caspase-1, Caspase-11, GSDMD), at 4 °C for 30 minutes, in dark. After washing twice, stained cells were analyzed using flow cytometry with CytExpert 1.1 software (Beckman Coulter, USA).

### Cell culture and *in vitro* stimulation

The murine macrophage cell line RAW264.7 was routinely cultured in Dulbecco’s Modified Eagle Medium (DMEM) complete medium supplemented with 10% fetal bovine serum (FBS) at 37 °C in a 5% CO_2_ incubator. Cells at the logarithmic growth phase were stimulated with uninfected red blood cell lysates (uRBC) or Plasmodium-infected red blood cell lysates (iRBC) at a cell-to-lysate ratio of 1:10. In selected groups, cells were pretreated with 200 ng/mL lipopolysaccharide (LPS) for 4 h, and the positive control group was stimulated with LPS combined with 10 μM nigericin.

### Assessment of pyroptosis phenotypes

Morphological changes in cells were observed under an inverted microscope, with characteristic ballooning of pyroptosis. Intracellular reactive oxygen species (ROS) levels were detected by flow cytometry using the DCFH-DA probe. Cell viability was assessed using the fixable viability dye (FVD). Lactate dehydrogenase (LDH) release into the culture supernatant was measured according to the manufacturer’s protocol to reflect the extent of damage to cell membrane integrity.

### Quantitative Real-Time PCR (qRT-PCR)

Total RNA was extracted from the isolated splenic cells and cultured cells using the TRIzol reagent, reverse-transcribed into cDNA, and then subjected to qRT-PCR using the SYBR Green kit. Relative gene expression levels were calculated using the 2^-^ΔΔCt method, with β-actin as the internal control. The genes examined included *Aim2*, *Ifi204*, *Asc*, *Casp1*, *Casp11*, *Gsdmd*, *Il1b*, and *Il18*. Primer sequences are listed in **Table 1**.

**Table 1:** Primer sequences used for qRT-PCR.

Gene	Forward primer (5′→3′)	Reverse primer (5′→3′)
Ifi204	CAGAAGTAACAGGAGAAACATCACT	GTTGCAGAAGTCTCGCCTCT
Aim2	AAATGCTGTTGTTGACCGGC	GAGTGTGCTCCTGGCAATCT
Il1b	TCTTTGAAGTTGACGGACCC	AGTGATACTGCCTGCCTGAA
Il18	TGGAGACCTGGAATCAGACAA	CTGGGGTTCACTGGCACT
Asc / Pycard	CCGAGTTTCATCCCCAGTCC	GCCTTTGATCCCAGGCCTAT
Casp1	GGACCCTCAAGTTTTGCCCT	GCAAGACGTGTACGAGTGGT
Casp4 / 11	TGTCTCATGGCACACTGCAT	GGCCTGCACAATGATGACTT
Gsdmd	TGGTGAAGCACGTCTTGGAA	TTTTCATCCCAGCAGTCCCC

### Western blot

Total protein was extracted from the isolated splenic cells and cultured cells, and proteins in the culture supernatant were enriched by trichloroacetic acid precipitation. Protein concentrations were determined using the bicinchoninic acid method. Equal amounts of protein were separated by sodium dodecyl sulfate-polyacrylamide gel electrophoresis and transferred onto polyvinylidene fluoride membranes. After blocking with 5% non-fat milk for 1 h, the membranes were incubated with primary antibodies against Caspase-1, Caspase-11, GSDMD, glyceraldehyde-3-phosphate dehydrogenase (GAPDH), and Tubulin overnight at 4 °C, followed by incubation with horseradish peroxidase-conjugated secondary antibodies for 1 h at room temperature. Protein bands were visualized using an enhanced chemiluminescence system.

### Statistical analysis

All data were analyzed using GraphPad Prism 8.0.1 software. Continuous data are presented as mean ± standard deviation. Comparisons between two groups were performed using an independent samples t-test, while comparisons among multiple groups were conducted using one-way analysis of variance(ANOVA). For data that did not follow a normal distribution, the Mann–Whitney U test was used. Normality was assessed using the Shapiro-Wilk test. Homogeneity of variance was evaluated using Levene’s test. For two-group comparisons, the unpaired Student’s t-test was used if normality and variance homogeneity were satisfied; otherwise, the Mann-Whitney U test was applied. For multiple-group comparisons, one-way ANOVA with LSD post-hoc test was used when assumptions were met. Error bars represent mean ± SEM. The exact number of biological replicates (n) is indicated in each figure legend (at least three mice per group for *in vivo* experiments; three independent experiments for *in vitro* assays). A *P* value < 0.05 was considered statistically significant.

## Results

### Up-regulation of pyroptosis-related gene expression in malaria patients

**Figure 1:**
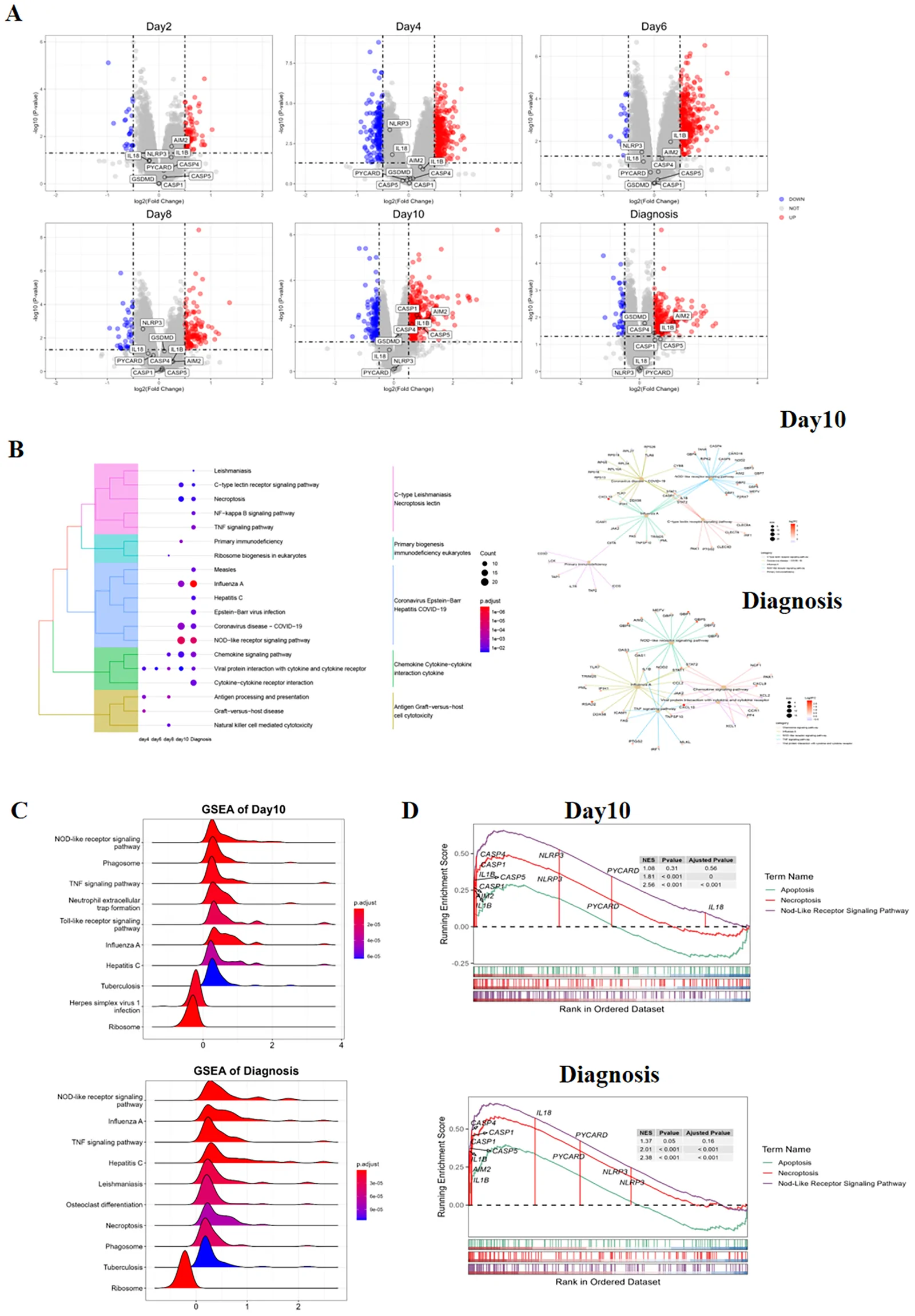
Transcriptomic analysis of pyroptosis-related genes in Plasmodium falciparum- infected patients. Analysis of whole blood transcriptome data from 19 individuals (15 volunteers experimentally infected with Plasmodium falciparum and 4 grouped by infection time (2, 4, 6, 8, and 10 days post-infection) and diagnosis. **(A)** The volcano plot shows the trend of changes in pyroptosis-related genes on different days of infection (differentially expressed genes [DEGs] defined by |log_2_(fold change)| > 0.5 and P < 0.05). **(B)** Kyoto Encyclopedia of Genes and Genomes (KEGG) enrichment analysis of differentially expressed genes in different groups. **(C)** Gene Set Enrichment Analysis (GSEA) analysis of the “Day 10” group and “Diagnosis” group. **(D)** Comparison of activation of apoptosis, necrosis, and pyroptosis in the “Day 10” group and “Diagnosis” group (statistical analysis performed using two-tailed unpaired t-test).

The GSE132050 dataset contains whole-blood transcriptomic data from 15 patients infected with *P. falciparum* and 4 healthy controls, with samples collected at sequential time points before infection and until malaria diagnosis. To explore the role of pyroptosis-related gene expression patterns in malaria patients, the GSE132050 dataset was analyzed. Differential expression analysis was performed for pyroptosis-related genes, including *GSDMD*, CASP1/4/5, *PYCARD*(ASC), NOD-like receptor family pyrin domain containing 3 (*NLRP3)*, *AIM2*, *IL1B*, and *IL18*. The results showed that the expression levels of these genes gradually increased during infection and peaked on the day of diagnosis (day 12), with statistical differences (*P <* 0.05, **Figure 1A**). KEGG pathway enrichment analysis revealed that the NOD-like receptor (NLR) signaling pathway was enriched from day 10 post-infection onward (**Figure 1B**). GSEA indicated that the pyroptosis gene set was activated in the infected group (**Figure 1C**). Comparison of different cell death pathways showed that both pyroptosis and necroptosis pathways were highly activated (*P <* 0.05), while the apoptosis pathway showed no obvious enrichment trend (**Figure 1D**).

### Co-activation of canonical and non-canonical pyroptosis-related genes in malaria patients.

**Figure 2:**
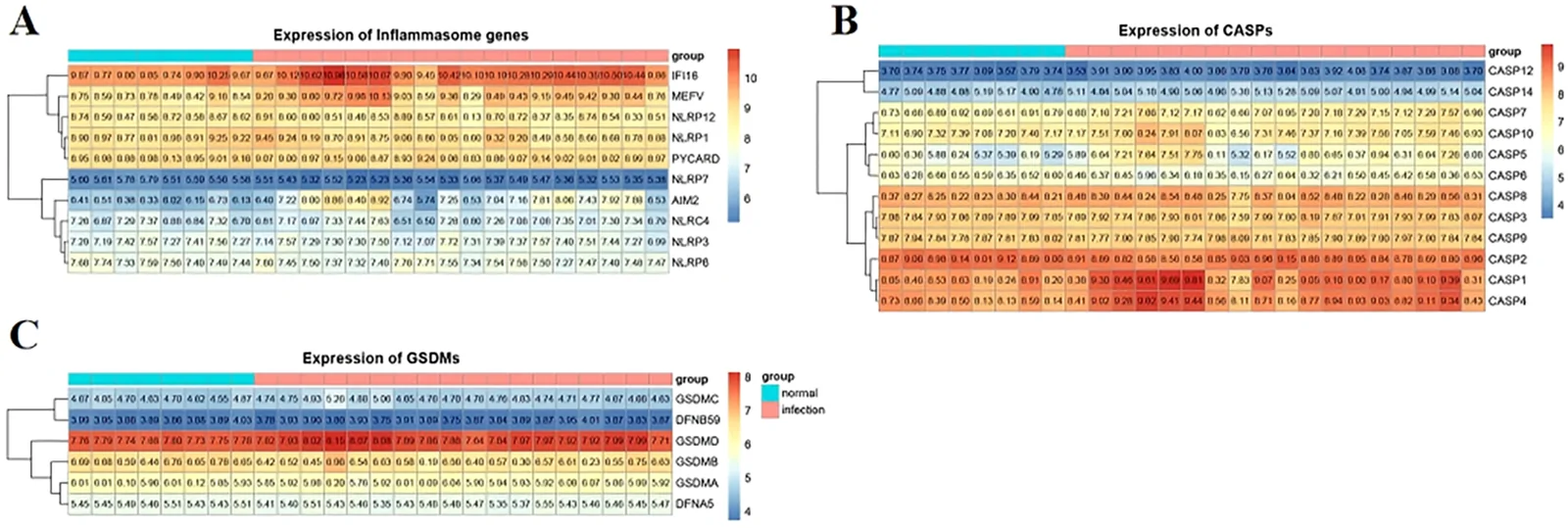
Plasmodium infection activates the classical and non-classical pathways of pyroptosis in patients. Heatmaps showing the expression of genes associated with pyroptosis before and after Plasmodium infection: **(A)** Inflammasome-associated genes. **(B)** Caspase gene family. **(C)** Gasdermin (GSDM) family genes.

Next, we compared the expression patterns of pyroptosis-related gene families at the time point of pyroptosis activation, including cytosolic pattern recognition receptors, caspases (CASPs), and gasdermins (GSDMs). Among the Gasdermin family, *GSDMD* exhibited the highest baseline expression level and showed a mild up-regulation after infection (**Figure 2A**). In the Caspase family, the expression levels ofCASP1 and CASP4/5 were significantly increased, while *CASP3*, *CASP8*, and *CASP9* showed no obvious changes (**Figure 2B**). Among the upstream receptors, *NLRP3* expression was only mildly altered, whereas *AIM2* and *IFI16* were evidently up-regulated after infection (*P <* 0.05; **Figure 2C**).

### Pyroptosis is highly correlated with myeloid cell expansion.

**Figure 3:**
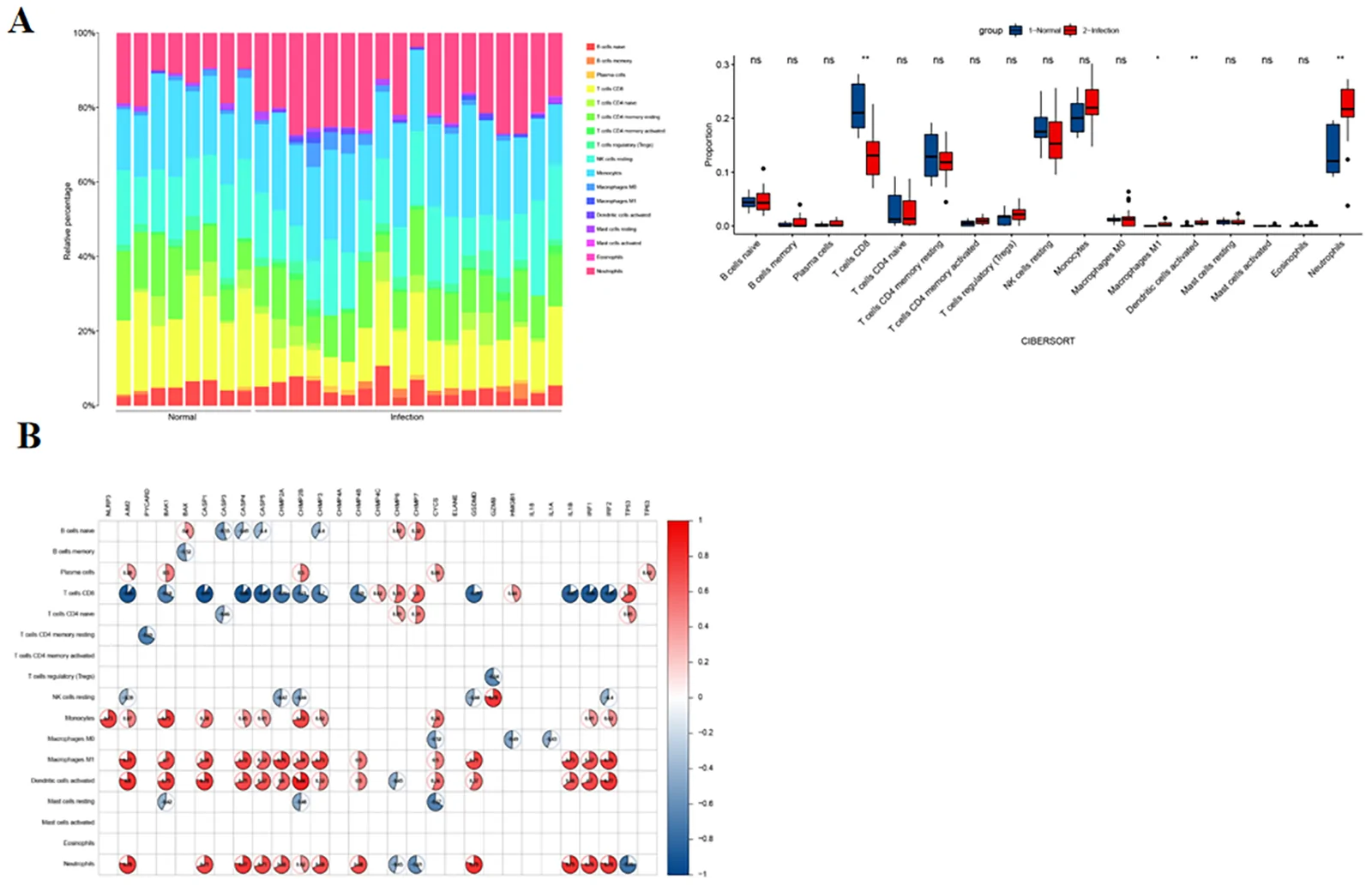
The induction of pyroptosis in malaria patients is most relevant to myeloid cells. Patient data (derived from 19 individuals (15 volunteers experimentally infected with Plasmodium falciparum and 4 healthy uninfected controls) were analyzed using CIBERSORT (Cell-type Identification By Estimating Relative Subsets Of RNA Transcripts) for immune infiltration analysis and Pearson correlation coefficients. **(A)** Immune infiltration analysis shows the relative proportion of immune cells in the whole blood of malaria patients. **(B)** Correlation analysis between the expression of pyroptosis-associated genes and immune cells. **P < 0.01, *P < 0.05, ns P > 0.05.

Furthermore, immune infiltration analysis showed that, compared with the pre-infection baseline, the proportions of peripheral blood monocytes/macrophages, dendritic cells, and neutrophils were significantly increased at the time of diagnosis (*P <* 0.05, **Figure 3A**). Correlation analysis revealed that the expression levels of core pyroptosis genes were positively correlated with the abundance of these myeloid cell subsets (**Figure 3B**).

### *Plasmodium yoelii* infection activates pyroptosis-related signaling in mouse splenic lymphocytes

**Figure 4:**
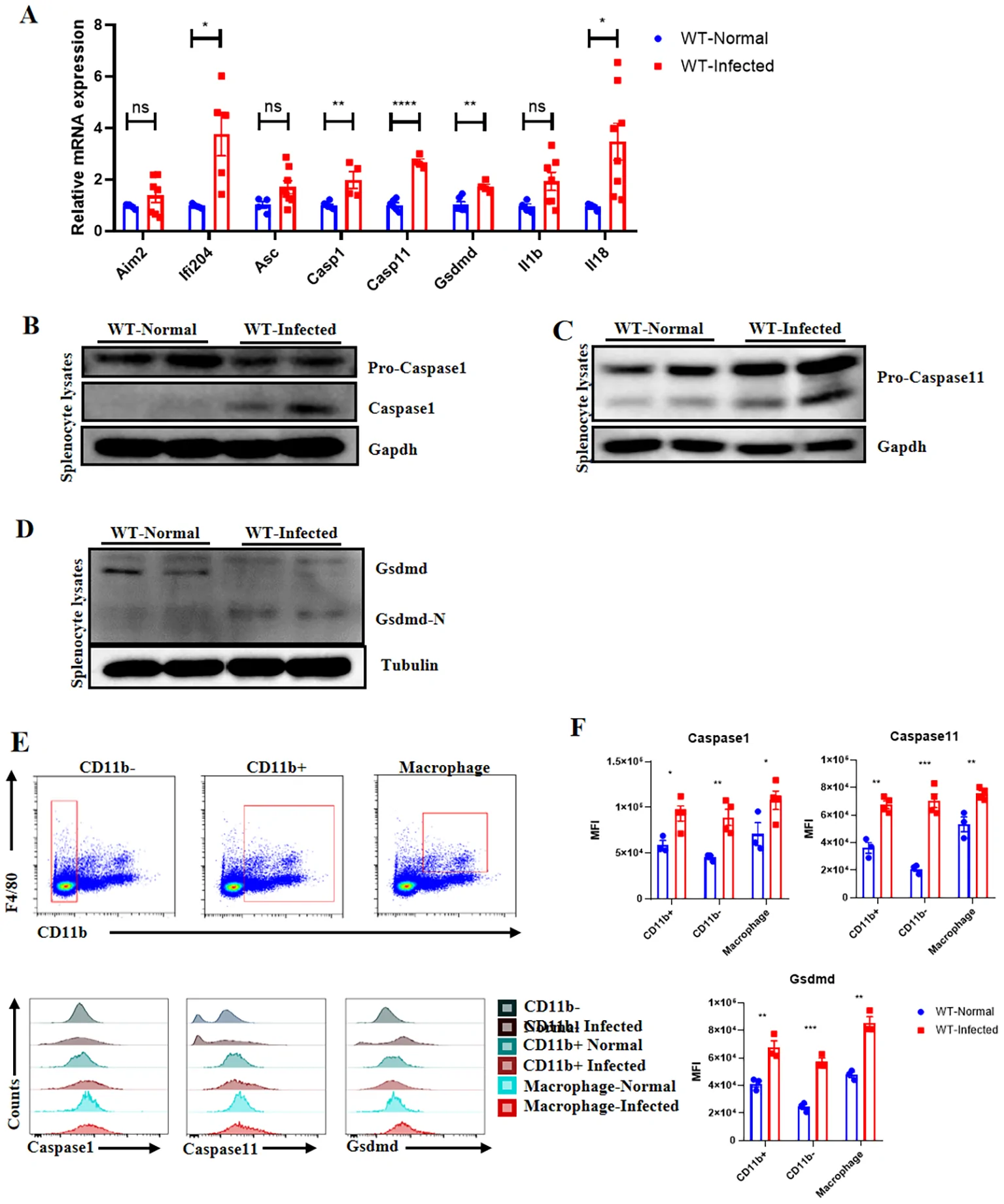
Plasmodium infection induces pyroptosis of splenic macrophages in mice. C57BL/6 mice were infected with Plasmodium by intraperitoneal injection, and the mice were killed 12 days post-infection. The spleens were collected for experiments. **(A)** Quantitative Real-time PCR is used to detect the expression of pyroptosis-related molecules in the spleen of Plasmodium-infected mice. **(B)** The expression of Caspase1, Caspase11, and GSDMD in splenocyte lysates from normal and infected groups is analyzed by Western Blot. **(C)** The diagrams and **(D)** statistical charts of Caspase1, Caspase11, and Gsdmd expression in CD11b-, CD11b+, and CD11b+F4/80+ cells detected by FACS. Data were performed in three independent experiments and presented as mean SEM. At least three mice per group were used in each independent experiment. ****P < 0.0001, ***P < 0.001, **P < 0.01, *P < 0.05, ns P > 0.05.

At the same time, C57BL/6 mice were infected with *P. yoelii.* On day 12 post-infection, splenic lymphocytes were isolated. Total RNA was extracted, and reverse-transcribed into cDNA for RT-qPCR. RT-qPCR. Results showed that the mRNA levels of *Aim2, Ifi204, Asc, Casp1, Casp11, Gsdmd, Il1*b, and *Il18* were significantly increased (*P* < 0.05, **Figure 4A**). Total protein was extracted from the isolated splenic cells, the concentrations were determined using the bicinchoninic acid method for western. Western blot analysis demonstrated that pro-Caspase-1 was cleaved into its active p20 fragment, and GSDMD was cleaved into the GSDMD N-terminal (GSDMD-N) active fragment in the spleen of infected mice. Pro-Caspase-11 expression was markedly up-regulated, but its cleaved active fragment (p26/p30) was not detected under our experimental conditions (**Figure 4B**). To identify the cellular source of these pyroptotic signals, flow cytometry was performed on splenic single-cell suspensions, revealing that the expression of Caspase-1, Caspase-11, and GSDMD was markedly increased in CD11b^+^ myeloid cells, with the highest expression intensity observed in CD11b^+^F4/80^+^ splenic macrophages (*P* < 0.05, **Figures 4C, D**).

### *Plasmodium* infected red blood cell lysates trigger pyroptosis-like responses and GSDMD cleavage in macrophages *in vitro*.

**Figure 5:**
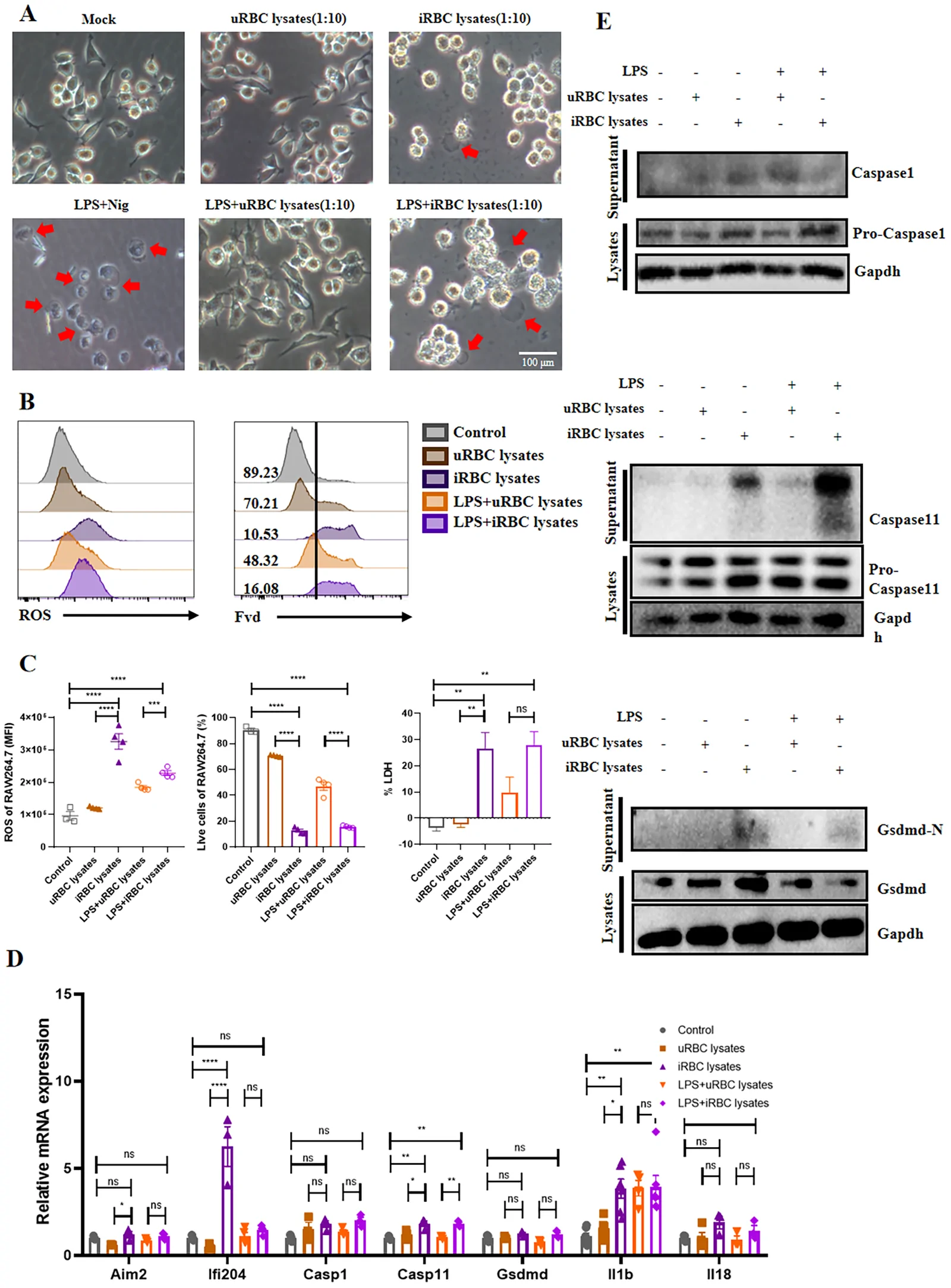
iRBC induces classical and non-classical pyroptosis pathways in macrophages in vitro. RAW264.7 was co-cultured with uRBC or iRBC lysates at a cell to RBC ratio of 1:10 for 48 h with or without LPS pre-stimulation for 4 h (200 ng/mL). **(A)** Morphological changes of RAW264.7 after different stimulation. LPS prestimulation for 4 h (200 ng/mL) followed by Nigericin (10 μM) stimulation for 3 h was used as a positive control. Images were taken using an inverted microscope. Scale bar: 100μm. **(B)** FACS diagrams of ROS and cell death detection. **(C)** Cell damage/death indicators (ROS/Fvd/LDH) statistical charts. Cells were collected for Quantitative Real-time PCR at 24h of stimulation **(D)**, and culture supernatants and cell lysates were collected for Western Blot at 48h of stimulation **(E)**. **(D)** Quantitative Real-time PCR is used to detect the expression of pyroptosis-related molecules in the iRBC-stimulated macrophage. **(E)** The expression of Caspase1, Caspase11, and Gsdmd in culture supernatants and cell lysates is analyzed by Western Blot. Data were performed in three independent experiments and presented as mean ± SEM. ****P < 0.0001, ***P < 0.001, **P < 0.01, *P < 0.05, ns P > 0.05.

After stimulation of RAW264.7 cells with iRBC lysates, microscopic examination revealed cell swelling and ballooning, and these morphological changes were exacerbated by LPS priming (**Figure 5A**). iRBC lysate stimulation led to increased intracellular ROS levels, decreased viable cell proportion, and significantly increased LDH release in the supernatant (*P* < 0.05, **Figures 5B, C**). RT-qPCR results showed that iRBC lysate stimulation induced the mRNA levels of Ifi204, Casp11, and Il1b (*P <* 0.05), while the transcript levels of *Aim2* and *Gsdmd* showed no significant changes (**Figure 5D**). Protein analysis revealed that active Caspase-1, active Caspase-11, and GSDMD-N were barely detectable in cell lysates but were markedly enriched in the culture supernatant (**Figure 5E**).

### R848 treatment suppresses the expression of pyroptosis-related proteins in splenic macrophages.

**Figure 6:**
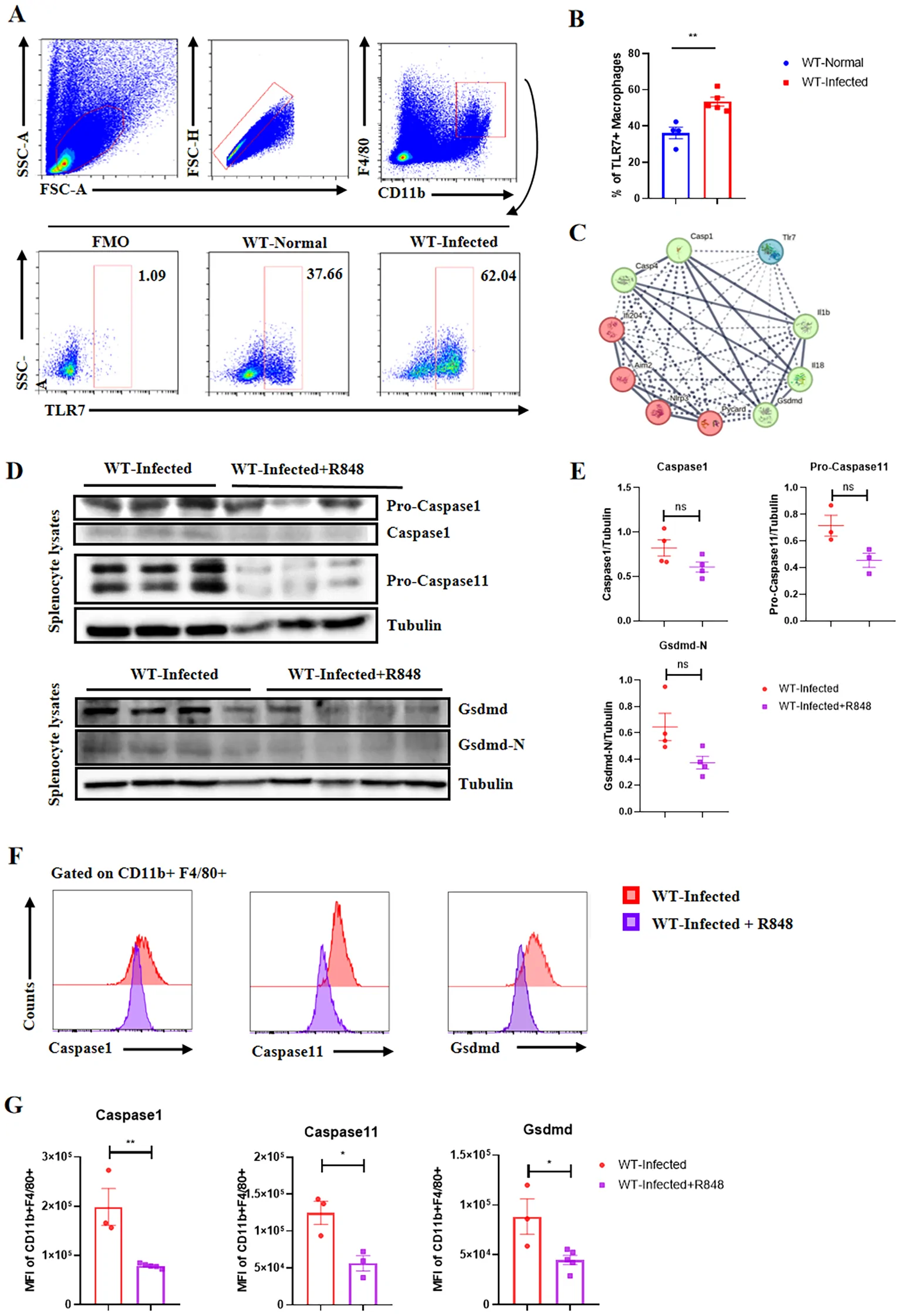
R848 inhibited the expression of pyroptosis molecules in splenic macrophages of Plasmodium-infected mice. Splenocytes from normal and infected mice were isolated 12 days post-infection, and the percentage of TLR7+ cells within the CD11b+F4/80+ population was measured by FACS. **(A)** FACS diagrams and **(B)** Statistical chart of TLR7 expression levels in splenic macrophages. STRING database was used to predict the relationship between TLR7 and pyroptosis molecules. **(C)** The correlation analysis between TLR7 and pyroptosis-related molecules. C57BL/6 mice were intraperitoneally infected with Plasmodium. Six days post-infection, the TLR7 agonist R848 was injected intraperitoneally at 100 μg per mouse, a total of 3 times. The infected mice were killed. On 12 days post-infection, Spleens from each group of mice were collected for the experiments. **(D)** Western Blot was used to detect the expression of key pyroptosis molecules (Caspase1, Caspase11, Gsdmd) in mouse splenocytes. **(E)** Gray-scale analysis of Western Blot results. **(F)** FACS diagrams of the expression of key pyroptosis molecules in splenic macrophages. **(G)** Statistical charts of the expression of key pyroptosis molecules in splenic macrophages. Data were performed in three independent experiments and presented as mean ± SEM. At least three mice per group were used in each independent experiment. *P < 0.05, **P < 0.01, ns P > 0.05.

After Plasmodium infection,Tlr7 expression was notably up-regulated in mouse splenic macrophages (*P <* 0.05, **Figures 6A, B**). STRING analysis indicated potential protein-protein interactions between TLR7 and key molecules of the pyroptosis pathway (**Figure 6C**). Following R848 intervention, the protein expression and activation levels of Caspase-1, Caspase-11, and GSDMD were reduced in the spleen of infected mice (*P <* 0.05, **Figures 6D, E**). Flow cytometry results demonstrated that R848 intervention significantly down-regulated the expression levels of Caspase-1, Caspase-11, and GSDMD in CD11b^+^F4/80^+^ macrophages (*P <* 0.05, **Figure 6F**). The inhibitory effect of R848 on Caspase-11 and GSDMD was clearly more potent than on Caspase-1 (*P <* 0.05, **Figure 6G**).

## Discussion

Pyroptosis is a lytic, pro-inflammatory form of programmed cell death driven by inflammatory caspases. In the context of infection, moderate pyroptosis can eliminate intracellular niches that harbor pathogen replication. However, excessive pyroptosis leads to massive release of pro-inflammatory cytokines, such as IL-1β and IL-18, as well as damage-associated molecular patterns, thereby inducing a systemic “cytokine storm” and severe histopathological injury (6, 19, 20). During the pathogenesis of malaria, this dysregulated inflammatory response is closely associated with the development of severe malaria, including cerebral malaria, severe anemia, and pathological splenic rupture (21, 22). Although previous studies have preliminarily confirmed inflammasome activation during Plasmodium infection, a systematic characterization of the canonical and non-canonical pyroptotic pathways in splenic macrophages, as well as their upstream regulatory mechanisms, remains largely lacking (23, 24). By integrating analyses of clinical transcriptomic cohorts, an *in vivo* Plasmodium *yoelii* infection model, and *in vitro* macrophage stimulation experiments, the present study not only demonstrates that Plasmodium infection is associated with activation of pyroptosis-related signaling pathways in macrophages, but also, our data provide evidence that further suggests that the TLR7 agonist R848 may exert an immunomodulatory effect during this process.

Deep bioinformatic analysis of the clinical whole-blood transcriptomic dataset (GSE132050) showed that, even at the pre-diagnostic stage of Plasmodium falciparum infection, the core pyroptosis gene set (GSDMD, CASP1/4/5, AIM2, and IFI16) exhibited progressive up-regulation as disease progressed. This feature was strongly positively correlated with the marked expansion of myeloid cell populations, particularly monocytes/macrophages. Clinical studies have indicated that aberrant activation of Caspase-1, Caspase-4/5, and GSDMD in peripheral blood monocytes may contribute to Plasmodium-induced immunopathological responses in macrophages (10, 16). In the murine *in vivo* model, we observed significant up-regulation of Aim2 and its mouse homolog Ifi204 (corresponding to human IFI16). AIM2 and IFI16, as key cytosolic DNA sensors, have been shown to recognize Plasmodium genomic DNA (gDNA) and hemozoin, thereby promoting assembly of the AIM2 inflammasome (9, 25). Our Western blot data further demonstrated that Caspase-1 and GSDMD were extensively cleaved into the active p20 and GSDMD-N fragments. In parallel, Pro-Caspase-11 was markedly elevated, but the absence of detectable cleaved active fragments suggests priming without full activation at the 12-day time point, rapid turnover of cleaved products, or technical limitations inherent to bulk tissue homogenates that may dilute or degrade labile active fragments. Moreover, FACS results indicated that the expression of Caspase-1, Caspase-11, and GSDMD was markedly increased in CD11b^+^F4/80^+^splenic macrophages. Consequently, robust biochemical evidence for non-canonical pyroptosis activation via Caspase-11 cleavage remains incomplete. Caspase-11 not only mediates pathogen-specific non-canonical pyroptosis (26, 27), but its excessive activation has also been linked to a complex cascade amplification effect involving acute hepato-splenic oxidative stress after erythrocyte lysis and macrophage necroptotic networks (28). These findings suggest that, in addition to NLRP3 and AIM2, the IFI16/IFI204-related canonical pathway and the Caspase-11-associated non-canonical pathway may contribute to Plasmodium-induced immunopathological responses in macrophages. Notably, GSEA also revealed concurrent enrichment of the necroptosis pathway (**Figure 1D**). LDH release, ROS production, and cell swelling are nonspecific indicators of inflammatory cell death and cannot distinguish pyroptosis from necrosis, secondary necrosis, or PANoptosis. Therefore, pyroptosis-specific validation (e.g., ASC speck imaging, Annexin V/PI staining, or electron microscopy) was not performed in this study and remains a future requirement.

At the *in vitro* level, stimulation of RAW264.7 macrophages with lysates of Plasmodium-infected red blood cells (iRBCs) faithfully recapitulated the canonical morphological hallmarks of pyroptosis, including cell swelling and membrane pore formation, as well as the pronounced release of ROS and LDH (29). Notably, we observed a seemingly paradoxical biological phenomenon in this experiment: substantial levels of active GSDMD-N and cleaved Caspase-1/11 were detected in the culture supernatant, whereas Gsdmd mRNA expression in the cells was not significantly elevated. From an immunological mechanistic perspective, this finding is highly consistent with the “two-signal” model of inflammasome activation (30, 31). As the executioner protein of pyroptosis, GSDMD is constitutively expressed at relatively high basal levels in resting macrophages and remains in an autoinhibited state (32). In the “two-signal” model of inflammasome activation, iRBC lysates serve as “Signal 2,” directly triggering the post-translational cleavage and activation of pre-existing Pro-Caspase-1/11 within the cell. Subsequently, the activated Caspase-1/11 rapidly cleaves the pre-existing GSDMD, which is in an autoinhibited state, releasing the N-terminal fragment (GSDMD-N) with pore-forming activity, ultimately causing membrane-targeted pore formation and a significant efflux of intracellular proteins (33, 34). Because pyroptosis is an acute and explosive form of cell death, its execution proceeds far more rapidly than a new round of transcription and translation. While this is consistent with the two-signal model, direct experimental validation of this model, such as time-lapse imaging of ASC speck formation and membrane pore kinetics, was not performed in this study, and passive release due to membrane rupture or extracellular vesicle shedding cannot be excluded as an alternative explanation for the accumulation of these proteins in the supernatant.

Consequently, activated proteins accumulated abundantly outside the cells and in the supernatant, whereas transcript levels remained largely unchanged. These findings support the possibility that Plasmodium-induced macrophage pyroptosis may involve a rapid post-translational response triggered by pathogen-derived components.

The most translational finding of this study is that the endosomal ssRNA receptor TLR7 was markedly up-regulated in infected macrophages, and intervention with the TLR7 agonist R848 not only failed to exacerbate inflammation, but instead significantly suppressed macrophage pyroptosis, with particularly strong inhibition of the Caspase-11/GSDMD non-canonical pathway. In classical immunological paradigms, TLR7 activation is generally known to induce type I interferon and pro-inflammatory mediators, and in certain bacterial infection models, IFN-1 is even a prerequisite for up-regulation of Caspase-11 (35, 36). However, within the complex Plasmodium infection network, receptor cross-talk is highly dependent on the microenvironment. Recent studies have shown that TLR7 activation in malaria models can polarize macrophages toward an M1 phenotype through the TLR7/signal transducer and activator of transcription 3 signaling axis, thereby markedly enhancing their ability to phagocytose and clear iRBCs (37). Meanwhile, moderate TLR7 activation can promote iron metabolism and extramedullary hematopoiesis in splenic macrophages, thereby preserving splenic structural and functional homeostasis (38). However, while R848 is commonly used as a TLR7 agonist, it may also engage TLR8-related pathways. As R848 was administered systemically *in vivo*, the observed suppression of pyroptosis may involve indirect mechanisms, such as type I interferon- or T cell-mediated effects, rather than direct macrophage-intrinsic immunomodulation alone. R848 can activate TLR7/TLR8-dependent MyD88 signaling and induce type I interferon production in multiple immune cell populations, which may secondarily alter inflammasome activity and IL-1β maturation (39–41). In addition, systemic TLR7/TLR8 stimulation may reshape antigen-presenting cell function and downstream T-cell responses, thereby indirectly modifying the inflammatory milieu surrounding splenic macrophages (42). Therefore, the reduced Caspase-1/11 and GSDMD signals observed after R848 treatment should be interpreted as the combined result of systemic immune regulation and macrophage-level changes, rather than as definitive evidence of a purely macrophage-intrinsic effect.TLR7-deficient mice were not included in the current R848 intervention experiments, therefore, the observed effects cannot be definitively attributed to TLR7-specific mechanisms without genetic validation. One possible explanation is that R848 may induce anti-inflammatory regulatory mediators, potentially including IL-10 or suppressor of cytokine signaling 1-related pathways, although this requires further experimental validation (8), which in turn feeds back to inhibit excessive inflammasome assembly, thereby establishing a new balance between “parasite clearance” and “avoidance of pyroptotic death.” By suppressing activation of pyroptosis-associated molecules, TLR7 signaling may rescue splenic macrophages from death, thereby ensuring their continued survival and functional competence as core phagocytic and antigen-presenting cells in antimalarial immunity (43).

Several limitations of the transcriptomic analysis should be noted: We acknowledge that these observations are correlative, genetic or pharmacological inhibition of GSDMD, Caspase-1, or Caspase-11 will be required to establish direct causality. Bulk whole-blood RNA-seq cannot assign gene expression to specific cell types, up-regulated pyroptosis transcripts do not equate to pyroptotic cell death, and the relatively small sample size (n=15) with longitudinal repeated measures warrants cautious interpretation. After multiple rounds of testing corrections, the threshold of *P* adj < 0.05 yielded only a small number of differential genes, which were insufficient for subsequent pathway enrichment analysis. Therefore, a threshold of *P* < 0.05 was chosen instead. We acknowledge that RAW264.7 is a transformed macrophage cell line and may not fully recapitulate primary macrophage biology, key *in vitro* findings should be validated in primary bone-marrow-derived macrophages in future studies.

In summary, this study demonstrates that Plasmodium infection is associated with activation of pyroptosis-related pathways in splenic macrophages *via* AIM2/IFI16/Caspase-1 canonical signaling and Caspase-11 non-canonical signaling. Targeted activation of the TLR7 signaling pathway by R848 exerts a critical negative regulatory effect, thereby limiting excessive pyroptosis. These findings expand our understanding of macrophage fate decisions during blood-stage malaria and suggest that modulating macrophage pyroptosis by targeting TLR7 signaling may represent a promising direction for future antimalarial immunomodulatory strategies.

## Data Availability

The datasets presented in this study can be found in online repositories. The names of the repository/repositories and accession number(s) can be found in the article/**Supplementary Material**.
